# Design, Fabrication and Characterization of a Low-Impedance 3D Electrode Array System for Neuro-Electrophysiology

**DOI:** 10.3390/s121216571

**Published:** 2012-12-03

**Authors:** Mihaela Kusko, Florea Craciunoiu, Bogdan Amuzescu, Ferdinand Halitzchi, Tudor Selescu, Antonio Radoi, Marian Popescu, Monica Simion, Adina Bragaru, Teodora Ignat

**Affiliations:** 1 Laboratory of Nanobiotechnology, National Institute for Research and Development in Microtechnologies (IMT-Bucharest), 126A, Erou Iancu Nicolae Street, 077190 Bucharest, Romania; E-Mails: florea.craciunoiu@imt.ro (F.C.); antonio.radoi@imt.ro (A.R.); marian.popescu@imt.ro (M.P.); monica.simion@imt.ro (M.S.); adina.bragaru@imt.ro (A.B.); teodora.ignat@imt.ro (T.I.); 2 Department of Biophysics and Physiology, Faculty of Biology, University of Bucharest, Splaiul Independentei 91-95, 005095 Bucharest, Romania; E-Mails: bogdan@biologie.kappa.ro (B.A.); fhalitzchi@yahoo.com (F.H.); tudorselescu@yahoo.com (T.S.)

**Keywords:** 3D electrodes, MEA, fabrication, electrochemical characterization, neuro-electrophysiology tests

## Abstract

Recent progress in patterned microelectrode manufacturing technology and microfluidics has opened the way to a large variety of cellular and molecular biosensor-based applications. In this extremely diverse and rapidly expanding landscape, silicon-based technologies occupy a special position, given their statute of mature, consolidated, and highly accessible areas of development. Within the present work we report microfabrication procedures and workflows for 3D patterned gold-plated microelectrode arrays (MEA) of different shapes (pyramidal, conical and high aspect ratio), and we provide a detailed characterization of their physical features during all the fabrication steps to have in the end a reliable technology. Moreover, the electrical performances of MEA silicon chips mounted on standardized connector boards via ultrasound wire-bonding have been tested using non-destructive electrochemical methods: linear sweep and cyclic voltammetry, impedance spectroscopy. Further, an experimental recording chamber package suitable for *in vitro* electrophysiology experiments has been realized using custom-design electronics for electrical stimulus delivery and local field potential recording, included in a complete electrophysiology setup, and the experimental structures have been tested on newborn rat hippocampal slices, yielding similar performance compared to commercially available MEA equipments.

## 1. Introduction

Over the last years, significant efforts have been directed towards developing structures for studies of neural networks, starting with small networks of cultured neurons [1]. Besides the essential information provided for a better fundamental understanding of the nature of cell response to different stimuli, and subsequently the manner of communication between them, these results should facilitate further developments of new therapeutic approaches. For instance, studying the simple visual stimuli effects on turtle retinal ganglion cells behavior helps to fabricate neuroprostheses based upon electrical stimulation of the retina, which represent a solution against profound blindness [2]. Recently, similar approaches were applied to other medically intractable or incurable diseases like Parkinson’s [3,4], Alzheimer’s [5–7], and even cancer [8,9], leading to significant improvements in the quality of life for these patients. In this perspective, the necessary studies on hippocampus for understanding information storage in the brain, learning and memory [10] are difficult via traditional methods such as the patch-clamp technique, because it is not easy to maintain a clamped neuron over hours, and often the assembly response of a neural network is more important than that of an individual cell.

Microelectronic sensors are the adequate choice for monitoring in- and output parameters of both cultured cells on neurochip surfaces or tissue slices brought in tight contact, and different integrated microelectrode array systems have been proposed by now [11,12]. Moreover, concerted research in development of miniaturized neural probes and probe arrays has led to the creation of micro-electronic mechanical systems generically named Neural MEMS or NeuroMEMS [13], strengthening the concept of “neuroscience-on-chip” [14]. In this context, microelectrodes, both arrayed and individually addressed, represent a solution for which the proof of concept has been demonstrated [15]. Although the interests in this area of microfabrication became known long time ago, there is still place for supplementary improvements, to find novel design solutions to make possible *in vivo* multiple sites recordings and allowing also a better sensitivity. Therefore, in order to ensure minimal signal attenuation and noise interference, it is critical to lower the interface impedance [16], which not only diminishes the recorded signals, but also requires more intense stimuli. Naturally, a solution is to have electronics to amplify and process signals in close proximity to bioelectrodes [17], but, although this approach is correct in its scope, it requires a high level fabrication technology (expensive, multilayer cleanroom processing), and consequently, the development of novel electrode technologies has to be appropriate to the general CMOS process flow of a complex electrical circuit, reducing the ability of varying the multi-electrode chip design.

Although in the last years novel materials, like metal-elastomer composite flexible conductor wires [18], carbon nanotubes [19], and new processes, even non-standard or unconventional, like metal transfer micromolding technology [20] or chemical polymerization [21] have been proposed to fabricate high performance bioelectrodes, silicon still remains the most used material, at least as substrate. Thus, the standard micromachining techniques allow multi-step silicon dry and wet etching followed by single or multiple metal deposition steps to define the necessary electrical circuits for electrodes contact, and furthermore, in terms of Si technology feasibility, it is clear that, due to its maturity and consequently reproducibility of the fabrication processes and ability of large scale implementation, it represents an alternative that cannot be ignored [22].

Besides the contact problems and forming of biohybrid interfaces, an important issue of all the proposed types of electrodes is their impedance. Given that low electrode impedance represents a figure of merit for sensitive detection, providing superior signal-to-noise ratio, highly necessary for noisy environments like neural electrophysiology applications, numerous studies have aimed to reduce impedance. It is inversely proportional to the surface area, but since for accurate point-like recording of local field potentials in neural networks an essential condition is using of low-diameter electrodes, the challenge resides, hence, in finding the most appropriate way to increase surface area of the electrode without increasing the diameter. Thus, one of our study goals was to quantify the impedance of the fabricated electrodes as a decisive test for appreciation of the design and technology reliability.

Gold nanofibers acting as close-packed nanoelectrode ensembles, carbon nanotube modified electrodes or platinum black coating have been used to increase the electrode surface. For instance, it has been shown that the impedance of the last type of electrodes has been lowered to a value of only ∼20 kΩ at 1 kHz for a 30 × 30 μm^2^ base area electrode [23], which is one of best achieved values, therefore we consider it as reference for our results. However, although the impedance of these systems is low, there are problems with the nanoelectrode quantification at stimulation/recording sites during electrophysiology studies, and consequently development of singular high-aspect ratio 3D-shaped electrodes is still under intensive investigation. This design, apparently simpler and with important advantages since the possible overlap of the radial diffusion layers from neighboring electrodes is eliminated, allowing also utilization as a bio-electrochemical tool [24], is not easy to be transferred in fabrication, and a compromise between highest aspect ratio figure implicitly yielding low impedance and the technological limitation should be attained.

Thus, starting from our previous experience in fabrication of microelectrode arrays for electrochemical sensors [25] and cell-based biosensors [26,27], we are going further to develop a novel silicon based multi-electrode array (Si-MEA) microchip that could meet the requirements for recording electrophysiological signals of neuronal networks at multiple points simultaneously. Accordingly, the objective of this paper is to present the proposed design and the MEMS technology processes particularly adapted to obtain superior microelectrodes, with a standard base area (30 × 30 μm^2^) and 3D shape, having the apex tip diameter of hundreds of nanometers. On the other hand, beyond experimental results related to system fabrication, electrochemical characterizations performed to assay the electrodes’ quality are also presented. Finally, preliminary tests performed on newborn rat hippocampal slices using the integrated micro-platform confirm that technologically we are on the right way, further neuro- electrophysiology studies being achievable.

## 2. Experimental Section

### 2.1. The Fabrication Process Flow for the Test Microchip

**Figure 1.**
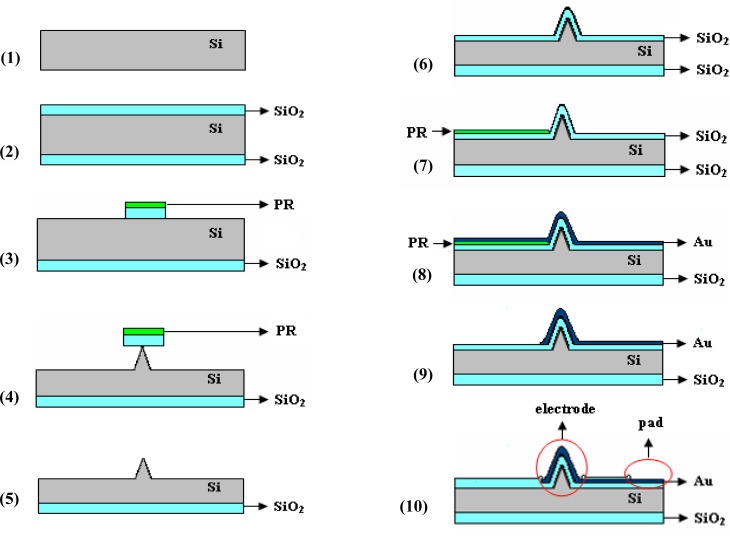
The fabrication process flow for the Si-MEA microchip.

The microchip proposed for neuro-electrophysiology applications was designed as an array of 25 individually addressed electrodes (a 5 × 5 matrix) coupled with a separate counter (signal ground or bath ground) planar microelectrode. The structure fabrication takes advantage of materials and processing tools of semiconductor and thin film technologies, exploring also novel solutions to obtain the most appropriate electrodes. The main steps of the process flow are: controlled etching of the silicon substrate to obtain 3D features corresponding to the potential electrodes, followed by substrate insulation, metallization/patterning of the electrical circuit, and ending by a new insulation which aims to maintain the metal accessible only on the electrodes and contact pads areas. Figure 1 presents a cross-section schematic view of the structure, focused on one electrode region, during these experimental stages.

Briefly, starting with a silicon (Si) wafer (*step 1*), a thin oxide (SiO_2_) layer (969 ± 1 nm in thickness measured by ellipsometry) was thermally grown to provide a masking layer for controlled Si etching (*step 2*). Next, a photolithographic process was used to pattern the substrate with 3D features: an approximately 1 μm-thick layer of positive photoresist (PR) was spun on the wafer, exposed and developed to mask (*Mask 1: Silicon etching*), finally resulting in areas of PR/SiO_2_ localized at the electrodes sites (*step 3*). They served as masking layers during the Si etching process (*step 4*), being removed by a short ultrasonic treatment when the contact area with the substrate was diminished to less than 1 μm^2^ (*step 5*). All these steps are generally required for fabrication of Si electrodes. Depending on the desired 3D shapes, additional processes can be used, and a more detailed discussion of our approach to obtain tall conical electrodes is presented in the next section.

A new oxidation process was used to electrically isolate the substrate, the measured thickness of this layer being 978 ± 1 nm (*step 6*). A critical step is metallization and the corresponding electrical circuit configuration [28], because a continuous and relatively smooth metal layer is preferred, even if high aspect ratio features are present on the substrate. Therefore, while metal deposition is realized by cathodic sputtering (100 nm Au with 10 nm Cr adhesion layer), which allows a conformal coating of patterned substrate, spinning of the photoresist masking layer is difficult, thus an excessively thick layer is necessary to be confident that it covers entire areas. Taking all these aspects into consideration, a lift-off process, where the resist is patterned before metal deposition using the transparent image of the electrical circuit photolithographic mask (*Mask 2: Electrical circuit*), is recommended (*step 7*). Consequently, the problematic regions of 3D features are exposed to UV light and should be photoresist-free, the layer continuity becoming less important. The metal deposition (*step 8*) and lift-off (*step 9*) followed. The last step for Si microchip fabrication was isolation of the conductive path to the electrodes, and several insulating passivation layers were tested, including different thicknesses of SU-8 negative-tone resist (*step 10*). The tests showed that a 992 ± 1 nm SiO_2_ insulating layer obtained by Plasma-Enhanced Chemical Vapour Deposition (PECVD) diminishes both the leakage capacitive and faradaic currents at satisfactory levels. The photolithographic process led to opening of the 3D-shaped electrodes and bonding pads (*Mask 3: Isolation*).

### 2.2. Microchip Interconnection, Assembling and Packaging

At the end of the microfabrication processes, the silicon wafer was sawed and the chips separated. Each of them was individually inspected by optical microscopy and SEM to eliminate those where different fabrication errors had happened (*i.e.*, lack of uniformity in the electrodes’ shapes or broken leads) and the best quality ones have been further prepared for tests. In order to provide control signal to the electrodes patterned on the silicon chip, this is mounted on a printed circuit board (PCB) where a 10 × 10 mm^2^ square-shaped cavity with the depth of 400 μm (approximately the Si chip thickness) is performed to overcome the difficulty of bonding the contact gold wires from gold pads to the gold-plated copper printed circuit. The PCB design was realized to plug into a standard 2 × 25 card edge connector (345/395), which has 10 mΩ maximum contact resistance.

The final step is gluing of a small plastic Petri dish (35 mm in diameter) with an 8 mm diameter hole drilled in the bottom to allow access to the electrodes, suitable for cell and tissue culture. The micro cell culture chamber is glued with silicone to the sensor chip mounted on the connector board, after filling the interspaced volume containing the bonding wires with similar material.

### 2.3. Electrochemical Characterization of the Test Electrodes

The scanning electrochemical microscope set-up (ElProScan, HEKA, Lambrecht, Germany) has been used to perform voltammetric analyses, with a current-sensitive preamplifier for high resolution low-noise recordings in the low pA range; also, experiments were carried out with chips located in a Faraday cage of aluminum sheets. Given that the test chips contain both the counter electrode and the array of individually addressable electrodes, the last ones served as the working electrode, the reference electrode being Ag/AgCl (KCl 3 M solution). Both linear sweep and cyclic voltammetry measurements have been carried out in 0.1 M KCl supporting electrolyte with/without probe molecule, 2 mM [Fe(CN)_6_]^3−^, using different potential sweep rates from 5 to 4,000 mV/s.

The evaluation of the electrode performance was further achieved by the full spectrum measurement of the electrode impedance magnitude. The same experimental set-up has been used for electrochemical impedance spectroscopy (EIS) analyses, except the potentiostat which has been replaced by PARSTAT 2273 (Princeton Applied Research, Oak Ridge, TN, USA). EIS was recorded over a frequency range of 100 Hz–500 kHz with logarithmic point spacing and potential amplitude of 10 mV rms at open circuit DC potential. The data have been fitted using the ZSimWin 3.21 software.

### 2.4. Electronics for Neuroelectrophysiology

For electrophysiology experiments we designed and built some custom electronic equipment in order to deliver electrical stimuli and collect local field potentials from tissue slices or cultured cells placed on the multi-electrode array (MEA) structure. The equipment was composed of six dual 8-channel CMOS analog demultiplexers, a series of high-performance JFET input operational amplifiers ensuring a 100-fold amplification, and a precision capacitive isolation amplifier for delivery of stimuli. The recording setup contained the above-described MEA device on the platform of a Bel Photonics STM Pro stereo zoom microscope (Bel engineering, Monza, Italy) equipped with a Bel 63X11H CCD camera connected to a computer via USB-2 interface. Solution was delivered to the Petri chamber under gravitational perfusion at a controlled flow rate of ∼6 mL/min, and the excess was drained to an expansion vessel connected to a vacuum pump. The electrical stimuli were delivered by an isolation electrophysiology stimulator (Isostim A320, WPI, Sarasota, FL, USA) triggered in EXT GATE mode by signals from the acquisition board. All these pieces of equipment were placed in a large Faraday cage. The electrical signals were converted with a Digidata 1440A interface controlled by the Clampex 10 software (Axon Instruments, Sunnyvale, CA, USA). We used either gap-free or episodic stimulation protocols with a sampling frequency of 10 kHz, applying either unique stimuli (0.1 ms duration) within 200 ms episodes, or stimuli preceded by conditioning pre-sweep trains (100 pulses/train, pulse frequency 500 Hz). In certain experiments the collected local field potentials were supplementary amplified 10-fold and hardware-filtered with a battery-operated DAM 50 differential amplifier (WPI).

### 2.5. Preparation of Brain Slices for Electrical Recording

We used male Wistar rats of age two to three weeks (P14–P21), kindly provided by the animal husbandry of the “N. Simionescu” Institute of Cell Biology and Pathology (Bucharest, Romania). All animal experiments were performed according to internal and international guidelines (EC Directive 86/609/EEC). Brain slices were prepared using custom procedures. 300 μm-thick sagittal slices were cut in ice-cold carbogen-saturated Ringer with a vibroslicer (MA752, Campden Instruments, Loughborough, UK), then stored in the same solution at room temperature. Adequate hippocampal sections were selected under a dissecting stereomicroscope, transferred in the recording MEA chamber, positioned with fine microtweezers and fixed with a heavy metal miniature horseshoe. The composition of the bicarbonate Ringer solution was the following (in mM): NaCl 115, KCl 5.6, MgCl_2_ 1, CaCl_2_ 2, NaHCO_3_ 25, NaH_2_PO_4_ 1, glucose 11 freshly added during the day of use. All chemicals were purchased from Sigma (St. Louis, MO, USA) or Merck (Darmstadt, Germany).

## 3. Results and Discussion

### 3.1. Microfabrication of the Test Structures

#### 3.1.1. Obtaining Different 3D-Shape Electrodes

**Figure 2.**
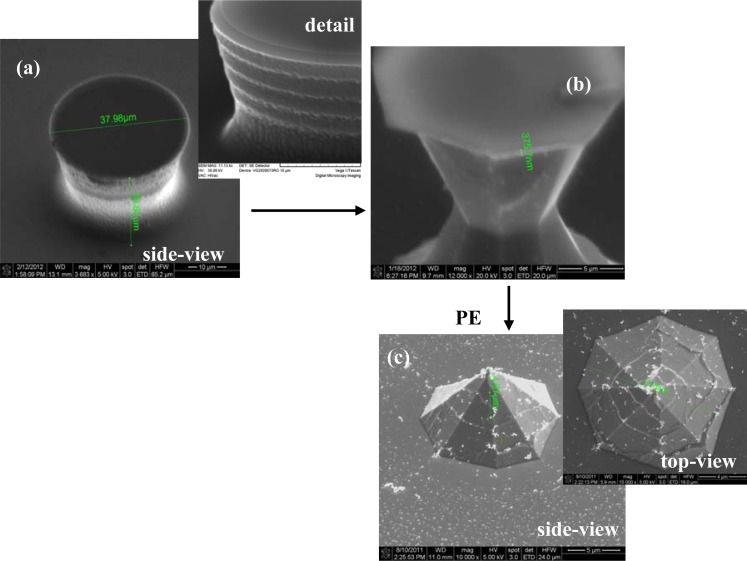
SEM images of an electrode subjected to dry (**a**) and anisotropic wet (**b**) etching processes.

**Figure 3.**
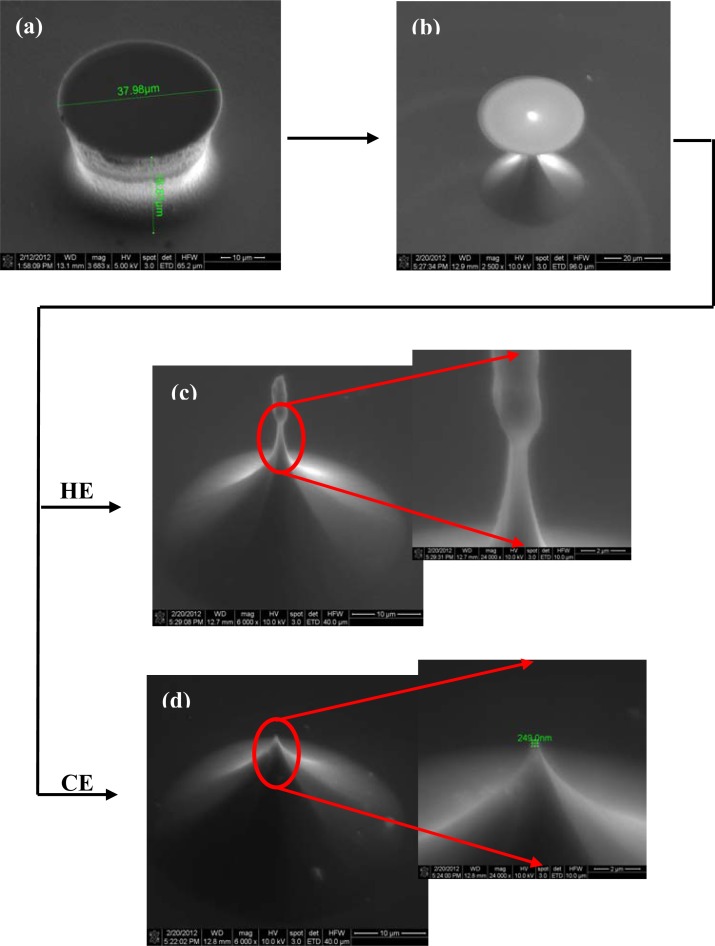
SEM images of an electrode in different stages of substrate dry and isotropic wet etchings.

The first critical step of Si microchip fabrication, important for its recording performances, is the controlled substrate patterning with the 3D electrode features. The wet etching process is commonly used for this purpose, comprehensive simulation and experimental studies of the profile evolution during anisotropic wet etching being already available [29]. Furthermore, additional technological processes can be utilized to improve the electrode characteristics; thus, the deep reactive ion etching (DRIE) leads to high aspect ratio electrodes [30] and also, a supplementary sharpening of the electrode tip could be achieved by using a short isotropic wet etching process [31]. Different etching routes have been explored, tuning the process parameters, to obtain the finest electrodes; actually, it was not a single process, because our technological experiments showed that better results are obtained when both dry and wet etching are used together. Accordingly, the substrate patterning was initiated by a time-multiplexed Bosch DRIE process using alternately 100 sccm C_4_F_8_ (100 W ICP, 10 W RF power levels; 30 mT pressure; 5 s) and respectively 100 sccm SF_6_ (100 W ICP, 25 W RF power levels; 30 mT pressure; 7 s) respectively, during 70 cycles. It took roughly 15 min to etch micropillars, with a height of ∼35 μm (Figures 2(a) and 3(a)). The measurement of the height of the pillar should be corrected with the tilted angle at which the picture was taken in the SEM (30°), giving a double real value.

Both types of wet etchings aimed sharpening the micropillars, starting with the most common one, the anisotropic process in 30% KOH solutions (Figure 2(b)). Consequently, 3D octogonal base pyramidal structures, determined by the crystal site-specificity of the etching rates at the atomistic level, have been obtained for a 5 minutes etching at 70 °C temperature. As the images show, the process is relatively rapid, starts from both sides of the micropillars (top and bottom), and the pyramidal structure height, and consequently the pyramidal electrode (**PE**) height is at less than half of the initial pillar. In addition, the stairs observed after dry etching are preserved during the wet process, a significant detail for the further accommodation of biological material. Alternatively, the isotropic process in the high oxidizing solution: HNO_3_-CH_3_COOH-HF in the volume ratio 25:10:1 (V/V/V), respectively, leads to smooth and taller conical structures as presented in Figure 3.

**Figure 4.**
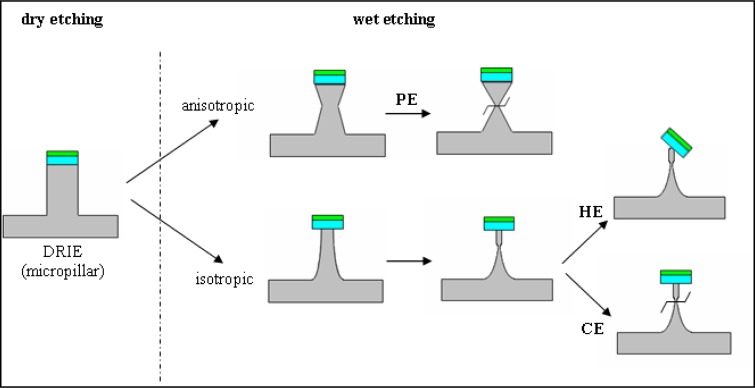
Schematics of the etching process for 3D electrodes of different shapes.

The structures have been regularly inspected during the etching, after each minute, to stop the process when the oxide caps (etch masks) remain hung up only by a small contact base, just before releasing (Figure 3(b)). There are two options identified at this point: (i) the first one is to apply a short ultrasonication treatment to remove the caps, obtaining high aspect ratio structures (and then high aspect ratio electrodes–**HE**); (ii) the second one is to continue the etching for few seconds, which results into further sharpening of the tips with consequent loss of height; however, they remain taller than the pyramidal structures (conical electrodes (**CE**)). Regarding the electrode apex tip diameters, they are around 150–250 nm for pyramidal (**PE**) and conical (**CE**) electrodes and 750–900 nm for the tallest ones (**HE**), respectively. Although the last type might not be as good in terms of penetrating tissue slices, its advantage is that, as well as the pyramidal terraced types, it is able to ‘automatically’ anchor the tissues [32]. Figure 4 presents schematically the evolution of 3D structures during different stages of the dry or wet etching processes.

The cross-section schematic view of one electrode during the substrate etching processes more clearly stresses the observed differences between them concerning the 3D-structure evolution. Accordingly, starting from similar DRIE fabricated micropillars, while the anisotropic etching commonly leads to pyramidal structures, the isotropic process allows fabrication of different profiles; due to the slow etching rate, it enables controlled undercutting of structures, and consequently more complicated features, from simple conical ones to long neck high aspect ratio ones. Therefore, the electrode height depends also on the selected etching process, being smaller than half of the initial pillar for anisotropic etching of pyramidal electrodes (**PE**), slightly bigger than half pillars (approximately two thirds) for conical electrodes (**CE**), and comparable for tall electrodes (**HE**), respectively. Consequently, their surface area directly depends on height, being the largest for **HE** types. It is worth mentioning that, although high aspect ratio features are obtained, the drawback of uniformity on whole structure is even more severe in this case, according to a delicate balance between stopping the etching at the right time and choosing the more appropriate ultrasonic power. Furthermore, they are also the most fragile during the lasting photolithographic processes. Therefore, structures with each of these three types of 3D elements have been subjected to the remaining processes from the proposed fabrication flow and further electrochemically characterized in order to decide on the most reliable technology.

#### 3.1.2. Fabrication of Electrical Circuit, Connecting and Packaging Test Structures

**Figure 5.**
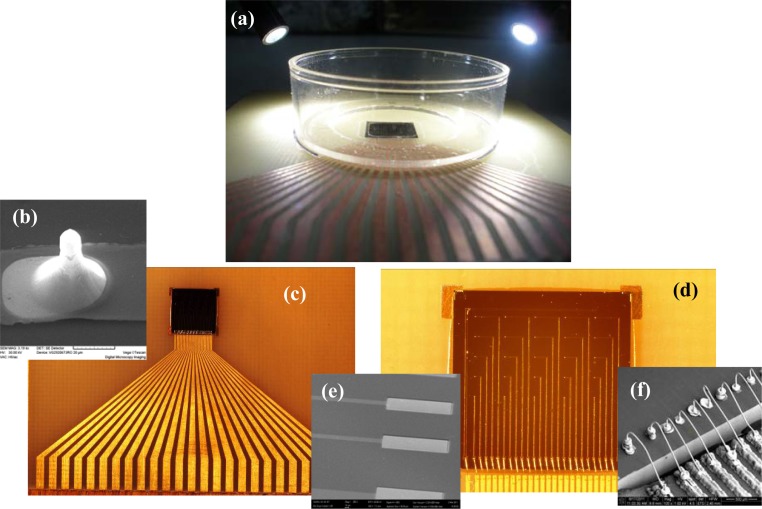
Photo-images of the final test structure (**a**) and respectively details of the PCB-mounted microchip (**c**,**d**); SEM images with details of one test electrode (**b**) and the microchip pads before and after their electrical connection with the PCB circuitry (**e**,**f**).

Following the technological steps described in the previous section, the etched Si substrate has been oxidized, metallized, photolithographically patterned, and insulated. Next, the microfabricated Si chip has been mounted and connected on PCB. Relevant images of the structures resulted during this process flow are presented in Figure 5.

Taking into account that the packaged system should remain intact for repetitive tests during several hours in a humid environment, one requirement for a successful assembly is to have the electrical contact from the electrode to the outside electronics protected by water-tight silicone. Moreover, as a supplementary measure of protection, after isolation of the bonded contact gold wires from gold pads to the gold-plated copper printed circuit, a small Petri dish with an 8 mm diameter hole drilled in the bottom to allow the access only to the electrodes was glued with silicone to the package—sensor chip and connector.

Figure 5(a) reveals the exposed area on the Petri dish (the dark colour part of microchip), rest of it being protected by silicone resin. A more clear view of the assembly selected for tests is shown in Figures 5(c,d). Details of different regions from this structure have been obtained using electronic microscopy. Thus in Figure 5(b) the image of one electrode can be identified and in Figure 5(e) the image of two pads for connecting recording electrodes (the upper ones) and the pad of bath ground electrode. In these SEM images, the exposed metal surfaces of the SiO_2_ insulated electrodes are clearly differentiated as the light-gray regions. Figure 5(f) represents an image of ultrasonically bonded gold wires. We have to mention that the continuity of the metal layer at the electrodes’ surface was confirmed by Energy-dispersive X-ray microanalyses (EDX). Subsequently, the final step was to verify the electrical connectivity using a multimeter, because the successful neurochip assembly is proved by the ability to maintain electrical contact from the electrode to the outside electronics. We have also confirmed that the mounting scheme is very robust, the test chips have survived several cycles of re-use over 1 year.

### 3.2. Electrochemical Characterization of 3D Electrodes

#### 3.2.1. Linear Sweep Voltammetry and Cyclic Voltammetry Investigation

**Figure 6.**
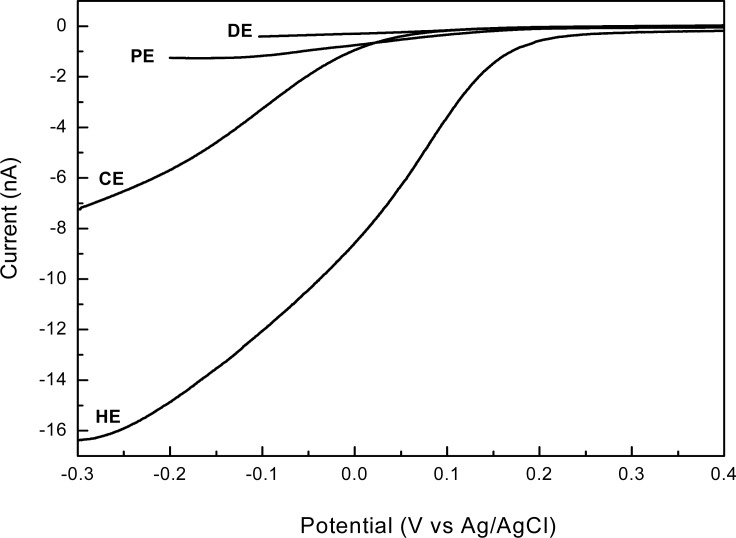
Linear sweep voltammograms recorded in presence of 2 mM [Fe(CN)_6_]^3−^ in 0.1 M KCl supporting electrolyte at 20 mV/s, using different types of electrodes: disk (**DE**); pyramidal (**PE**); conical (**CE**); and high aspect ratio (**HE**).

To assess the electrochemical performances of the newly developed 3D shaped electrodes, we compared their responses with those of a standard planar disk shaped electrode (**DE**). Voltammetric measurements, both linear sweep and cyclic, are valuable tools when investigating limiting currents and electroactive area of electrodes. The linear sweep voltammograms, obtained in presence of 2 mM [Fe(CN)_6_]^3−^ in 0.1 M KCl supporting electrolyte, using each type of electrodes—disk electrode (**DE**); pyramidal electrode (**PE**); conical electrode (**CE**) and high aspect ratio electrode (**HE**)—are presented in Figure 6.

As shown in this figure, different sweeping ranges were adopted in our study: while for planar electrodes the diffusion-limited current was established around −0.1 V, by increasing the electrode area it reached more negative values, around −0.2 V, and finally extended to −0.3 V for **CE** and **HE** types. A plateau region extended over a large potential range indicates that the limiting current value is not affected by the mixed control region of the primary reaction and the current generated from the secondary reaction. This is the case of **DE** and **PE**. In some cases, however, the potential range of the plateau region is less than 0.2 V and a poorly defined plateau region may exists when both primary and secondary reactions occur at similar potentials or where the mixed control region extends close to the potential of the secondary reaction [33]—this may be the case of **CE** and **HE**. On the other hand, the linear steady state diffusion-limited currents are in good agreement with SEM images, a more than 40-fold increase in current being obtained by fabrication of a high aspect ratio 3D electrode (**HE**), which has the same base with those of planar disk electrodes (**DE**). Accordingly, the measured currents determined by each type of electrode are: 0.4 nA **(DE)**, 1.25 nA **(PE)**, 7.0 nA **(CE)** and 16.25 nA **(HE)**, respectively, being a confirmation of the strong dependence of the magnitude of limiting currents on the electrode electroactive areas at a given concentration of ferricyanide.

**Figure 7.**
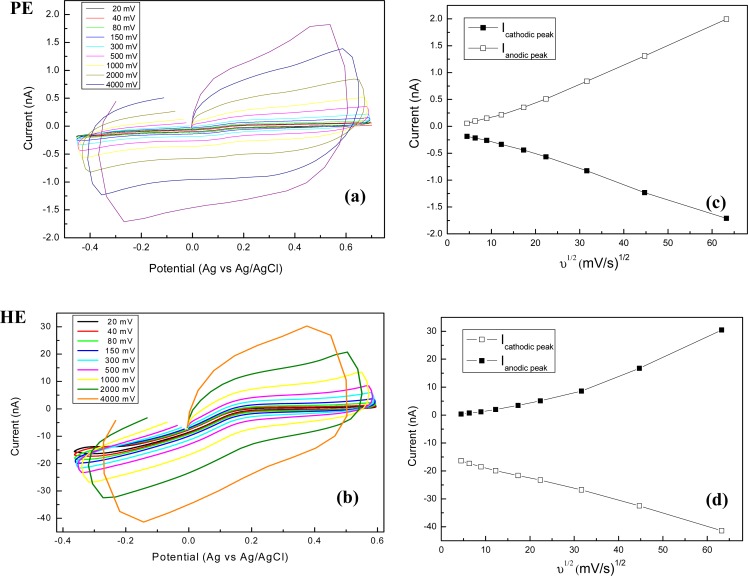
Cyclic voltammograms in 2 mM Fe(CN)_6_^3−^ in 0.1 M KCl, recorded at different scan rates using **PE** (**a**) and **HE** (**b**) types electrodes and the corresponding dependence of cathodic/anodic peak currents on the square root of the sweep rates (**c**,**d**).

The cyclic voltammograms recorded for pyramidal (**PE**) and high aspect ratio (**HE**) electrodes at different scan rates, from 20 mV/s to 4 V/s, are presented in Figure 7(a,b). Furthermore, an analysis of scan rate dependences is realised as plots of the cathodic/anodic peak currents versus the square root of the sweep rates in Figure 7(c,d).

The voltammograms in Figure 7, showing a symmetrical sigmoidal response, are different when compared with classical peak-shaped ones, specific for macroelectrodes. In our case, the linearity of current *vs.* square root of scan rate translates a linear relationship typical for a diffusion-controlled process. For **PE** electrodes the correlation coefficient was 0.9991 for both anodic and cathodic sweeps, meanwhile the linearity was comprised between 80 and 4,000 mV/s. For **HE** electrodes the correlation coefficient was 0.9986 for the potential range of anodic sweep at cycling rates of 20–500 mV/s and 0.996 for the cathodic sweep at all rates (20–4,000 mV/s), respectively.

#### 3.2.2. Electrochemical Impedance Spectroscopy

**Figure 8.**
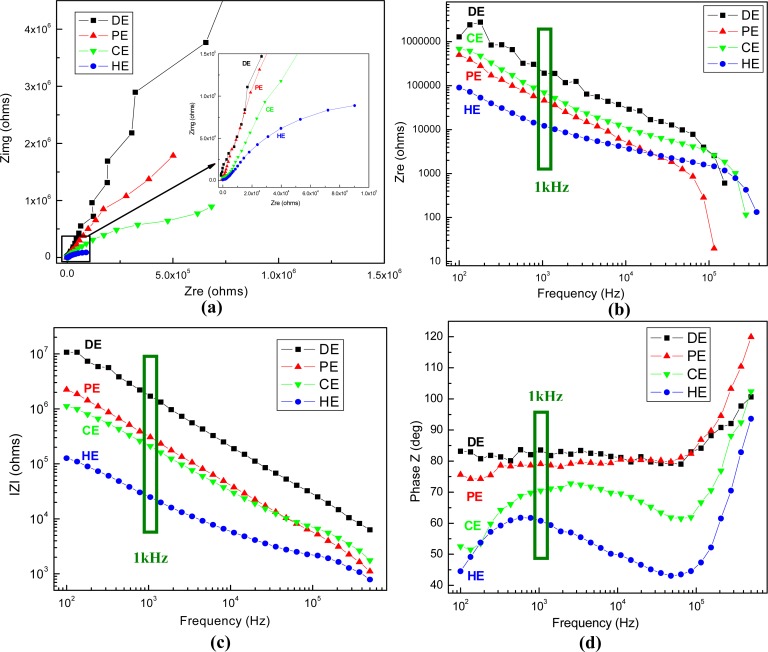
Impedance response of different electrodes—**DE**, **PE**, **CE**, **HE**—in electrolyte solution of 2mM Fe(CN)_6_^3−^ in 0.1 M KCl at OCP: (**a**) complex impedance plane (Nyquist plot); (**b**) real part of the impedance, (**c**) its modulus and (**d**) the phase shift as functions of frequency (Bode plots).

The physico-chemical properties of the electrode-analyte interface have been further characterized by electrochemical impedance spectroscopy technique. The impedance responses of all fabricated types of gold plated electrodes at open circuit potential (OCP) are presented in Figure 8.

The graphs show that the results obtained for the four types of electrodes are easily differentiated in the impedance plots, which represent transfer functions characteristic of parallel RC passive filters. The non-linearities at higher frequencies may represent structural rearrangements of mobile charge carriers and/or surface charge density variations at the interface due to local electrical field inhomogeneities. At frequencies of ∼100 kHz (for the PE electrode) or higher, the phase shift reaches −90°, indicative of a purely capacitive response, and above this point the conductance increases sharply, in a process suggestive of the loss of dielectric constant occurring in ice-like water microdomains. The equivalent circuit generally assigned to the present electrochemical system contains three main elements, the resistance of solution, the capacitance of electrical double layer at the electrode interface and the resistance of charge transfer, and it has been used to fit the Nyquist plots with ZSimWin software. An important element is the electrolytic capacitor formed by the metal-electrolyte interface, which has frequency dependence, and takes a value about 0.2 pF/μm^2^ at 1 kHz, the frequency of interest for neuronal action potentials. The impedance values recorded at 1 kHz for each type of electrodes are summarized in Table 1.

The results indicate that the impedance modulus features the lowest values for the high aspect ratio electrodes (**HE)**, being more than sixty-fold lower compared with the planar ones (**DE**), from MΩ to kΩ. If the latest reported values of the electrodes acceptable for cell-based sensors are ranging in general between a few tens of kΩ to 1 MΩ [34,35], the best achieved impedance value being ∼20 kΩ for the Pt black-coated electrode of similar base area, our **HE** type electrode has impedance on the order of 20–30 kΩ, which is an excellent value to provide accurate data for the envisaged applications. On the other hand, while the phase is above −70° for the first three types, demonstrating capacitive impedance, it becomes −60° for **HE**, which might indicate both capacitive coupling and resistive conduction for signal transmission [36].

Furthermore, taking into account that the envisaged application of the proposed structures is extracellular recording of the action potentials, which are usually “spikes” in the noisy extracellular signal, implying in fact a phase of “spike detection”, the analysis of noise sources becomes significantly important. Thus, the Johnson’s noise rms (standard deviation) magnitude is given by Nyquist’s formula:

(1) $$V(\mathrm{rms})={\left(4k_{B}\mathrm{TR}\Delta f\right)}^{1/2}$$

where ***k_B_*** = 1.38 × 10^−23^ J/K is Boltzmann’s constant, ***T*** is the absolute temperature (room temperature 300 K), ***R*** the electrode resistance (Ω), and ***Δf*** the bandwidth (Hz).

Given that the measured real part of the 1 kHz electrode impedance varies between 205 kΩ for plane disk electrodes and 12 kΩ for tall electrodes, the corresponding noise level varies between 5 μV and 1 μV, which represents a five-fold decrease. It is important to mention that, while for extracellular stimulation the level of noise is less critical, typical applied voltages being of the order of volts, the amplitude of extracellularly-measured neuronal spikes is over four orders of magnitude lower, often of tens of μV [12,37]. In this view, the supplementary reduction of electrode resistance represents a significant achievement.

### 3.3. Preliminary Tests on Newborn Rat Hippocampal Slices

**Figure 9.**
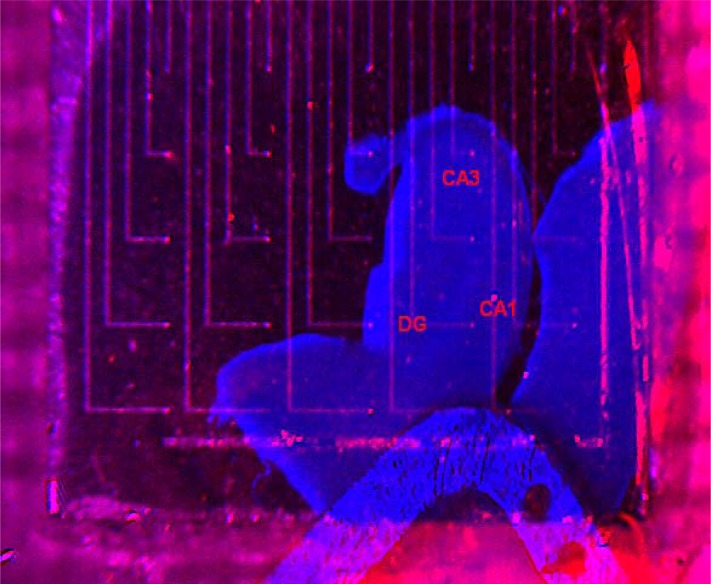
Synthesis image of a newborn rat (P18) 300-μm hippocampal slice placed over the MEA, fixed with the miniature metallic horseshoe; several regions of interest are marked (DG – dentate gyrus, CA3 and CA1).

The 300-μm thick hippocampal slices did fit very well on the microelectrode array and could be arranged such that certain regions of interest (e.g., the dentate gyrus, CA3, CA1) were placed over specified electrodes. At the indicated superfusing solution flow rate the slices remained stable over longer time intervals, if properly fixed with the miniature metallic horseshoe. Figure 9 represents an artificially colored overlap image constructed in ImageJ from individual frames taken with the stereomicroscope camera before and after placement of the preparation, allowing precise identification of electrode positions relative to the slice anatomy and selection of an appropriate pair of electrodes for stimulation and recording.

**Figure 10.**
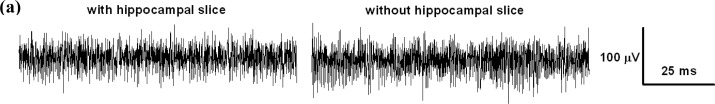

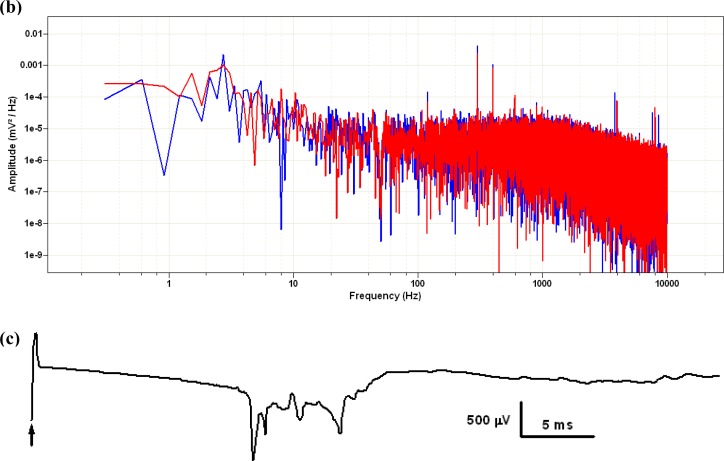
Short stretches of recording with or without slice preparation placed on the MEA. (**a**) unfiltered signal; (**b**) their Fourier transform spectra (blue: with slice; red: without); (**c**) stimulus-evoked series of local field potentials (stimulus application marked with arrow).

The electrical behavior of the MEA device during recordings was adequate. The noise level of the unfiltered signal sampled at 10 kHz did not exceed 100 μV peak-to-peak and could be further reduced by appropriate hardware or software filtering. In Figure 10 we represented two brief (100 ms) intervals of recording taken with or without the preparation in place, and the corresponding power spectra of longer (10 s) stretches of the same recordings obtained via Fourier transform in Clampfit10, after applying a 50-Hz electrical interference removal soft filtering. When stimuli were applied between pairs of electrodes in the absence of the biological preparation we could record passive artifacts containing both conduction signals and capacitive artifacts.

According to the selected pair of electrodes, their amplitude was generally correlated with the position of the recording electrode in relation to the average conduction path in the bath electrolytic solution between the stimulating and reference electrode. When a slice was present, we could elicit and record stimulus-triggered electrical activity.

## 4. Conclusions/Outlook

A novel fabrication process flow has been proposed to obtain Si-based multi-electrode array chips with individually addressed 3D electrodes of various shapes, essentially achieved by fine-tuning of the Si etching methods and their related parameters. The experimental structures have been carefully microscopically analyzed during all the fabrication steps to have in the end a reliable technology for each type of structures, with pyramidal (PE), conical (CE) or high aspect ratio (HE) electrodes.

Moreover, the MEA structures mounted on gold-plated PCB connectors were non-destructively electrochemically tested using impedance spectroscopy and both linear sweep and cyclic voltammetry to characterize the electrode activities. Thus, the linear steady-state diffusion-limited currents are in good agreement with surface details as revealed in SEM images, a more than 40-times increase in current being obtained by fabrication of a high aspect ratio 3D electrode (HE), which has the same base with those of planar disk electrodes (DE). Also, the voltammograms show symmetrical sigmoidal responses, which are different when compared with classical peak-shaped ones, specific for macroelectrodes. On the other hand, the EIS results indicate that the impedance modulus features the lowest values for the high aspect ratio electrodes (HE), more than sixty-fold lower compared to the planar ones (DE), from MΩ to kΩ, being in the range of 20–30 kΩ, on the order of the best reported ones and thus, stimulation currents up to ±500 μA could be delivered.

Further, mounted on gold-plated PCB connectors, they were included in custom-designed and built equipment in view of use within neuro-electrophysiology experiments on tissue slices or cultured cellular networks. Preliminary tests on acute newborn rat hippocampal slices yielded satisfactory results, similar to those previously reported using similar devices and types of experiments. Given the relative accessibility of silicon-processing technology and flexibility in design and manufacturing, we conclude that this class of MEA devices represents a promising development in several areas of fundamental and applied biomedical research.

## Figures and Tables

**Table 1.** The values of impedance module, real part and imaginary part, phase shift and capacitance measured for the electrodes at 1 kHz frequency.

***1 kHz***	**IZI** (kΩ)	**Phase** (°)	**Zre** (kΩ)	**Zimg** (kΩ)

**DE**	1,659.6	−81.69	204.63	200.27
**PE**	323.1	−79.97	71.55	30.85
**CE**	217.3	−70.50	47.18	29.94
**HE**	26.8	−60.79	12.22	4.47
